# Successful resuscitation from prolonged hypothermic cardiac arrest without extracorporeal life support: a case report

**DOI:** 10.1186/s13256-019-2282-6

**Published:** 2019-12-02

**Authors:** Melanie Kuhnke, Roland Albrecht, Joerg C. Schefold, Peter Paal

**Affiliations:** 1 Swiss Air Rescue, Swiss Air-Ambulance (Rega), P.O. Box 1414, 8058 Zurich, Switzerland; 2 Department of Intensive Care Medicine, Inselspital, Bern University Hospital, University of Bern, Bern, Switzerland; 3 Department of Anesthesiology and Intensive Care Medicine, Hospitallers Brothers Hospital, Paracelsus Medical University, Salzburg, Austria

**Keywords:** Hypothermia, Cardiac arrest, Cardiopulmonary resuscitation, ECLS, Mechanical CPR

## Abstract

**Background:**

We report a case of successful prolonged cardiopulmonary resuscitation (5 hours and 44 minutes) following severe accidental hypothermia with cardiac arrest treated without rewarming on extracorporeal life support.

**Case presentation:**

A 52-year-old Italian mountaineer, was trapped in a crevasse and rescued approximately 7 hours later by a professional rescue team. After extrication, he suffered a witnessed cardiac arrest with ventricular fibrillation. Immediate defibrillation and cardiopulmonary resuscitation were started. His core temperature was 26.0 °C. Due to weather conditions, air transport to an extracorporeal life support center was not possible. Thus, he was rewarmed with conventional rewarming methods in a rural hospital. Auto-defibrillation occurred at a core temperature of 29.8 °C after 5 hours and 44 minutes of continued cardiopulmonary resuscitation. With a core temperature of 33.4 °C, he was finally admitted to a level 1 trauma center and extracorporeal life support was no longer required. Seven weeks following the accident, he was discharged home with complete neurological recovery.

**Conclusions:**

Successful rewarming from severe hypothermia without extracorporeal life support use as performed in this case suggests that patients with primary hypothermic cardiac arrest have a chance of a favorable neurological outcome even after several hours of cardiac arrest when cardiopulmonary resuscitation and conventional rewarming are performed continuously. This may be especially relevant in remote areas, where extracorporeal life support rewarming is not available.

## Background

Cardiac arrest from severe accidental hypothermia is a rare event. The clinical outcome may be favorable if patients are resuscitated in time and rewarmed until return of spontaneous circulation (ROSC) [1]. During the past two decades, extracorporeal life support (ECLS) rewarming has become a cornerstone in the treatment of patients with hypothermia-induced cardiac arrest [2, 3]. We report the case of a patient in whom ECLS rewarming was not possible due to bad weather impeding transport to an ECLS center. Cardiopulmonary resuscitation (CPR) was performed for 5 hours and 44 minutes and the patient rewarmed with warm forced air, warm blankets, and warm infusions only. Auto-defibrillation occurred. After 20 days in an intensive care unit and 7 weeks in hospital, the patient was discharged neurologically intact.

## Case presentation

**Fig. 1:**
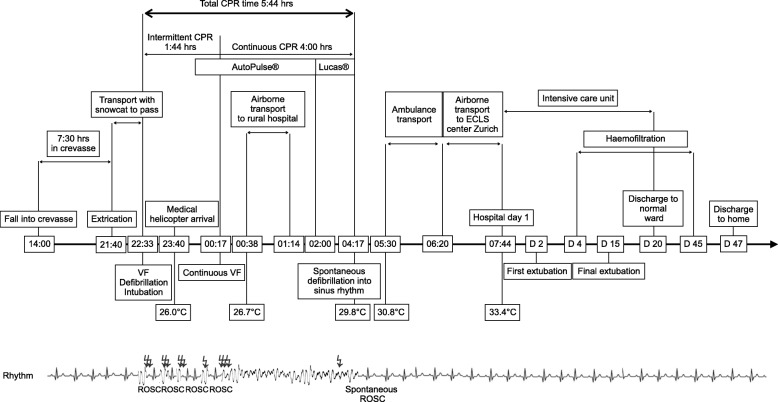
Timeline of rescue and further course. *CPR* denotes cardiopulmonary resuscitation, *ECLS* extracorporeal life support, *hrs* hours, *ROSC* return of spontaneous circulation, *VF* ventricular fibrillation, *lightning image* defibrillation

A 52-year-old lightly dressed Italian mountaineer was hiking alone on an Italian glacier at 3085 m, when he fell about 15 m into a crevasse at approximately 14:00 (Fig. 1). Ice-water was pouring over him, but he was able to breathe. His wife alerted a rescue team to report him missing. After over 7 hours in the crevasse, he was found and rescued at 21:40. He complained about being cold but was in no pain. Then he lost consciousness. Night had fallen and low clouds prevented a helicopter rescue. Thus, extraction using terrestrial mountain rescue with a snowcat was commenced. He remained unconscious but was spontaneously breathing; oxygen saturation was 84% with 2 L oxygen/minute through a face mask at a heart rate of 60/minute. His trachea was not intubated at this stage because the mountain rescuers on site were only basic life support certified.

At 22:33, after arrival at a mountain pass at 2750 m, he developed ventricular fibrillation (VF), which initially responded to immediate defibrillation, but then recurred. ROSC was achieved two more times with a total of six defibrillations; his blood pressure was 120/70 mmHg. During resuscitation, he was intubated. A Swiss rescue helicopter with night-flying capability was requested to transport the patient directly to an ECLS rewarming center. When the helicopter arrived at 23:40, his esophageal temperature was 26.0 °C. An electrical warming blanket (Geratherm® UniqueResc+, Geratherm Medical AG, Geschwenda, Germany) was placed on his abdomen, and a mechanical chest compression device was placed in case of VF recurrence.

Soon after, VF recurred and was terminated with one defibrillation, but after a few minutes, VF recurred again. Three further defibrillation attempts remained unsuccessful; thus, continuous CPR with mechanical chest compression (AutoPulse®, Zoll Medical, Cologne, Germany) was started, further defibrillation attempts were considered obsolete at a core temperature of 26.0 °C [3]. Weather conditions again precluded transport to the ECLS center and he was air transferred to the closest rural hospital. An arterial blood gas analysis showed a pH of 7.2, lactate 11.2 mmol/L, partial pressure of oxygen in arterial blood (paO_2_) 18.1 kPa, and potassium 2.7 mmol/L. This encouraged the team to continue CPR and conventional rewarming, and to push for either transport of the patient to an ECLS center or for a portable ECLS device to be transferred to the patient. In the meantime, the mechanical chest compression device was exchanged with a LUCAS®, because it featured a plug-in power supply (LUCAS®, Jolife AB, Physio Control, Lund Sweden). Conventional rewarming was performed with forced warm air (Bair Hugger®, 3 M, St. Paul, MN, USA) over his abdomen and inferior extremities and intravenously with warmed normal saline. Warm cotton blankets were placed on his chest around the LUCAS® device and over his head and changed every 15–30 minutes. The net rewarming rate was 0.7 °C/hour.

After a total of 5 hours and 44 minutes of CPR with 4 hours of mechanical CPR and at a core temperature of 29.8 °C, conversion into sinus rhythm occurred spontaneously with sustained ROSC. Finally, at 5:30 a.m., transport by terrestrial ambulance was undertaken across a mountain pass. At 6:20 a.m., weather conditions allowed transport by air to the nearest ECLS center (Zurich, Switzerland). On arrival, his core temperature was 33.4 °C, sinus rhythm. By then, ECLS rewarming was no longer required.

On the second day after admission to the ECLS center, he was awake with a Glasgow Coma Scale of 15. Respiratory and renal failure required mechanical ventilation and continuous hemofiltration*,* followed by intermittent dialysis with good recovery of renal function. He was ventilated for 12 days, extubated on day 15 and discharged from the intensive care unit at day 20. Seven weeks after the accident, he was discharged home from rehabilitation with complete neurological recovery despite a persisting neuropathy in his feet and hands. Four months after the accident, he resumed his job.

## Discussion

Several cases of hypothermic cardiac arrest with successful CPR and favorable neurological outcome were previously reported [2, 3]. However, this case seems remarkable for many reasons. First, this is the longest documented case of CPR with survival after hypothermic cardiac arrest and successful rewarming without ECLS, pleural lavage or peritoneal lavage [4, 5]. In addition, this is one of the longest cases of successful CPR (5 hours and 44 minutes), 4 hours of which were performed using a mechanical CPR device [6]. Second, in the past two decades, successful resuscitation with non-ECLS rewarming has become less common as ECLS rewarming is encouraged in current resuscitation guidelines [3, 7, 8] following a landmark study [2]. This case confirms, however, that successful rewarming of cardiac arrest from severe hypothermia is possible by non-ECLS means in cases in which a functioning rescue chain with a dedicated team and continuous CPR are provided. Oxygen demand decreases 6–7% per 1 °C of cooling [9, 10]. In normothermia, CPR states with such durations are not survivable without neurological damage.

For patients with a core temperature < 30 °C with persistent VF, the advanced cardiovascular life support (ACLS) algorithm of the American Heart Association states that it may be reasonable to perform further defibrillations and administer vasopressors (class IIb) [11]. In contrast, the European Resuscitation Council Guidelines recommend a maximum of three defibrillations and no vasopressors [8]. This discrepancy can be explained by the different interpretation of mainly animal data which show that vasopressors increase the chances of successful defibrillation < 30 °C but ROSC is not stable and tends to degrade into VF again.

If a patient remains asystolic after rewarming, death most likely occurred (for example caused by asphyxia) before the onset of hypothermic cardiac arrest [3]. Moreover, in hypothermic cardiac arrest, a serum potassium > 12 mmol/L indicates a low probability of good outcome and discontinuation of CPR should be considered [3, 11]. In accidentally hypothermic patients in cardiac arrest, serum potassium is a prognostic marker [12–14].

In this case the principles of both Advanced Life Support (ALS) and Advanced Trauma Life Support (ATLS) guidelines were applied.

## Conclusions

In conclusion, we report a case of successful non-ECLS-based rewarming of a patient with witnessed primary hypothermic cardiac arrest with complete neurological recovery. Extensive CPR was provided making this case one of the longest cases of CPR in the literature that resulted in a favorable neurological outcome. In patients with primary hypothermic cardiac arrest, successful rewarming can thus also be achieved with continuous CPR being provided in the absence of ECLS systems. This case should encourage medical teams in their efforts in providing continuous CPR in primary hypothermic patients even without available ECLS.

## Data Availability

The datasets analyzed in the case report are available from the corresponding author on request.

## Abbreviations

CPR: Cardiopulmonary resuscitation
ECLS: Extracorporeal life support
ROSC: Return of spontaneous circulation
VF: Ventricular fibrillation
